# Prevalence and changing distribution of HTLV-1 and HTLV-2 infections in Spain

**DOI:** 10.1186/1742-4690-8-S1-A86

**Published:** 2011-06-06

**Authors:** Ana Treviño, Antonio Aguilera, Estrella Caballero, Rafael Benito, Patricia Parra, Jose M Eiros, Araceli Hernandez, Enrique Calderón, Manuel Rodríguez, Alvaro Torres, Juan García, Jose Manuel Ramos, Lourdes Roc, Goitzane Marcaida, Carmen Rodríguez, Matilde Trigo, Cesar Gomez, Raul Ortíz de Lejarazu, Carmen de Mendoza, Vincent Soriano

**Affiliations:** 1 Hospital Carlos III, Madrid, Spain; 2 Hospital Conxo, Santiago de Compostela, Spain; 3 Hospital Vall dÂ´Hebron, Barcelona, Spain; 4 Hospital Clínico Universitario Lozano Blesa, Zaragoza, Spain; 5 Hospital Clínico Universitario, Valladolid, Spain; 6 Hospital Insular, Las Palmas de Gran Canaria, Spain; 7 Hospital Universitario Virgen del Rocío, Seville, Spain; 8 Hospital Universitario Puerta del Mar, Cádiz, Spain; 9 Hospital Universitario de Canarias, Santa Cruz de Tenerife, Spain; 10 Hospital Cristal-Piñor, Orense, Spain; 11 Hospital General, Elche, Spain; 12 Hospital Miguel Servet, Zaragoza, Spain; 13 Hospital General Universitario, Valencia, Spain; 14 Centro Sanitario Sandoval, Madrid, Spain; 15 Complejo Hospitalario, Pontevedra, Spain; 16 Complejo Hospitalario Virgen de la Salud, Toledo, Spain; 17 ana.trevino.rc@gmail.com

## Background

Most HTLV-1/2 infections in Spain have been found in native intravenous drug users carrying HTLV-2. However, the large immigration flows from Latin America and Africa during the last decade may have changed the prevalence and distribution of HTLV-1 and HTLV-2 infections, and hypothetically even open the opportunity for introducing the new HTLV-3 or HTLV-4 variants.

## Methods

A national multicenter, cross-sectional, national study was conducted in June 2009 recruiting consecutive adult outpatients attending 16 different hospitals in Spain. HTLV-1/2 antibodies were screened using an EIA and further confirmed using a commercial Western blot. Samples with indeterminate Western blot patterns were examined using specific PCR assays for discriminating HTLV types 1 to 4.

## Resuts

A total of 6,460 patients were studied. Overall, 40% were males and 88% were native Spaniards. The main origin of the immigrant population was Latin America (4.9%), Africa (3.6%) and other European countries (2.8%). A total of 9 individuals were seroreactive for HTLV antibodies (overall prevalence 0.14%). Of these, Western blot confirmed as HTLV-1 in 4 (prevalence 0.06%) and HTLV-2 in 5 (prevalence 0.08%). Another 2 specimens yielded EIA reactivity close to the cut-off and could not be confirmed as positive for any of the HTLV types 1 to 4. All but one HTLV-1 cases were Latin Americans while all HTLV-2 cases were native Spaniards. The single HTLV-1 subject born in Spain was a 31 year-old woman who denied any significant risk behavior for HTLV exposure, including intravenous drug use, sexual promiscuity, transfusions or travel to Latin America. All HTLV-1 carriers but one were asymptomatic. A woman from the Dominican Republic was diagnosed with mild TSP/HAM.

## Conclusions

The overall prevalence of HTLV-1/2 infections in Spain remains low, although a shift in the relative proportion of HTLV-1 and HTLV-2 seems to have occurred in recent years. The recognition of HTLV-1 in a native Spaniard with lack of recognizable risk factors for HTLV-1 exposure should alert about a spread of HTLV-1 outside classical risk groups. No cases of HTLV-3 nor HTLV-4 infections have been identified so far in Spain.

